# Molluscan benthic communities at Brijuni Islands (northern Adriatic Sea) shaped by Holocene sea-level rise and recent human eutrophication and pollution

**DOI:** 10.1177/0959683618788651

**Published:** 2018-08-02

**Authors:** Sara-Maria Schnedl, Alexandra Haselmair, Ivo Gallmetzer, Anna-Katharina Mautner, Adam Tomašových, Martin Zuschin

**Affiliations:** 1 Department of Palaeontology, University of Vienna, Austria; 2 Earth Science Institute, Slovak Academy of Sciences, Slovak Republic

**Keywords:** benthic community, conservation paleobiology, death assemblage, historical ecology, molluscs, sediment core

## Abstract

The effects of and the interplay between natural and anthropogenic influences on the composition of benthic communities over long time spans are poorly understood. Based on a 160-cm-long sediment core collected at 44 m water depth in the NE Adriatic Sea (Brijuni Islands, Croatia), we document changes in molluscan communities since the Holocene transgression ~11,000 years ago and assess how they were shaped by environmental changes. We find that (1) a transgressive lag deposit with a mixture of terrestrial and marine species contains abundant seagrass-associated gastropods and epifaunal suspension-feeding bivalves, (2) the maximum-flooding phase captures the establishment of epifaunal bivalve-dominated biostromes in the photic zone, and (3) the highstand phase is characterized by increasing infaunal suspension feeders and declining seagrass-dwellers in bryozoan-molluscan muddy sands. Changes in the community composition between the transgressive and the highstand phase can be explained by rising sea level, reduced light penetration, and increase in turbidity, as documented by the gradual up-core shift from coarse molluscan skeletal gravel with seagrass-associated molluscs to bryozoan sandy muds. In the uppermost 20 cm (median age <200 years), however, epifaunal and grazing species decline and deposit-feeding and chemosymbiotic species increase in abundance. These changes concur with rising concentrations of nitrogen and organic pollutants due to the impact of eutrophication, pollution, and trawling in the 20th century. The late highstand benthic assemblages with abundant bryozoans, high molluscan diversity, and abundance of soft-bottom epi- and infaunal filter feeders and herbivores represent the circalittoral baseline community largely unaffected by anthropogenic impacts.

## Introduction

Investigations on modern marine ecosystem responses to natural and anthropogenic changes are typically limited to annual or decadal time scales. However, marine ecosystems have experienced major human impacts over the past few hundred years (Jackson et al., 2001; Lotze et al., 2006), and consequences of such impacts are difficult to capture using ecological data, which are limited to a few years or decades (Kidwell, 2015). Death assemblages (DAs) of shelly fauna preserved on the seafloor or in sediment cores provide a window to the past (Edgar and Samson, 2004; Gallmetzer et al., 2017). They allow us to reconstruct community composition before and after the onset of human interference, and to assess community responses both to long-term natural environmental changes and human impacts (Gilad et al., 2018; Mautner et al., 2018; Rick et al., 2016). Therefore, the investigation of DAs can identify baseline community states that can serve as a reference point for management and restoration of sensitive, endangered marine areas (Dietl et al., 2015; Jackson et al., 2001; Kidwell, 2013; Rodriguez et al., 2001).

The northern Adriatic Sea is a prime example of an ecosystem strongly affected by centuries of human pressure (Barmawidjaja et al., 1995; Gallmetzer et al., 2017; Huntley and Scarponi, 2015; Kowalewski et al., 2015; Lotze et al., 2011; Mautner et al., 2018; Scarponi et al., 2017a). This semi-enclosed shallow basin with high riverine input, extensive soft bottoms, susceptibility to seasonal water-column stratification, and propensity to eutrophication due to high primary production in its western part is classified as particularly sensitive (Stachowitsch, 1984) and is considered one of the most degraded marine ecosystems worldwide (Lotze et al., 2006). Benthic mortalities caused by oxygen depletion and mucilage events have occurred here periodically for centuries (Crema et al., 1991; Danovaro et al., 2009; Tomašových et al., 2017) but became more frequent and widespread during the last 40 years (Degobbis et al., 2000; Faganeli et al., 1985; Justić, 1991; Stachowitsch, 1991). The increase in the frequency of hypoxia was promoted by anthropogenic eutrophication through agriculture and wastewater disposal (Barmawidjaja et al., 1995; Mavrič et al., 2010; Nerlović et al., 2011; N’siala et al., 2008; Vidović et al., 2016). Overfishing and pollution negatively affected ecosystem functioning, leading to changes in food-web structure and to reduced efficiency of bentho-pelagic coupling (Barausse et al., 2009; Coll et al., 2007). Exploitation by fishermen started early in history in the Neolithic age approx. 4000 BC (European Commission, 2017) as fish, mussels and shellfish were popular trading goods and important food sources for early settlers (Forenbaher and Miracle, 2005; Horvat, 2015; Jackson et al., 2001). Harbour, ship traffic and insufficient waste water management are the main causes for pollutants in the northern Adriatic Sea (Howarth, 2008; Pearson and Rosenberg, 1978). However, in spite of extensive paleoecological research in the northern Adriatic Sea, the Holocene history of shallow-shelf benthic ecosystems off the Istrian peninsula along the NE Adriatic shelf, which are associated with very low sedimentation rate, remains poorly known (Mautner et al., 2018).

In this study, we use molluscan DAs from a sediment core collected at 44 m water depth to reconstruct the history of the north-eastern Adriatic macrobenthic fauna off southern Istria since the Holocene transgression (~11,000 years ago). First, we hypothesize that large-scale environmental processes (i.e. sea level rise and changes in sediment input and turbidity) lead to long-term changes in species composition and diversity of molluscan assemblages, as well as in the sedimentary record of pollutants and organic matter. Second, we assess whether human impacts can be detected in the stratigraphic record, and suggest that the most rapid decrease in diversity and total molluscan abundance occurs in the youngest core layers that correspond to the past few centuries.

## Material and methods

### Study area

The northern Adriatic was flooded ~11,000 years ago at sites presently located at ~40 m (assuming subsidence of ~0.8 mm/year, Antonioli et al., 2009) and ~10,000 years ago at sites located at 30–35 m water depth (Antonioli et al., 2007; Storms et al., 2008; Vacchi et al., 2016), and maximum ingression was attained in the NW Adriatic ~7000–6000 years ago (Amorosi et al., 2016, 2017). Its surface circulation is primarily thermohaline and cyclonic (counterclockwise), with the south-eastward Western Adriatic Current in large part driven by the Po River, the largest river in the area and the main source of freshwater, sediments, and nutrients (McKinney, 2007). The productivity in this shallow sea (average water depth 35 m) is among the highest in the generally oligotrophic Mediterranean (Zavatarelli et al., 1998). The basin is characterized by a west-eastern gradient in nutrients (from eutrophic to oligotrophic) and sediment composition (from fine-grained muds to coarse-grained bioclastic sediments) (Zuschin and Stachowitsch, 2009). Holocene sediment cores from the north-eastern Adriatic show a succession from fluvial to brackish and marine facies (Cattaneo and Trincardi, 1999; Orogelec et al., 1991). High concentrations of Hg, Cu and Zn characterize modern deposits that accumulated during the past thousand years in the Gulf of Trieste (Covelli et al., 2006; Mautner et al., 2018; Vidović et al., 2016). Temporary sediment storage and resuspension mainly occur during the winter storm period caused by north-easterly cold winds (bora), but also during strong summer winds from southeast (sirocco) (Frignani et al., 2005). Sediments are resuspended by bottom trawling and dredging (Kaiser et al., 2002; Thrush and Dayton, 2002), which occurred in the Adriatic Sea for more than a century (De Madron et al., 2005; Palanques et al., 2001). Moreover, due to water column stratification from summer to mid-autumn, short-term hypoxic and anoxic events, frequently coupled with mucilage events, causing mass mortalities of benthic communities occur frequently in the northern Adriatic Sea (Crema et al., 1991; Stachowitsch, 1991; Tomašových et al., 2017). These events tend to occur at almost annual frequency in the NW Adriatic off the Po Delta (Alvisi and Cozzi, 2016). However, they also affect the Gulf of Trieste and NE Adriatic off Istria, although with smaller frequency and less regularly (Djakovac et al., 2015; Harding et al., 1999).

### Sampling and sediment analysis

**Figure 1.**
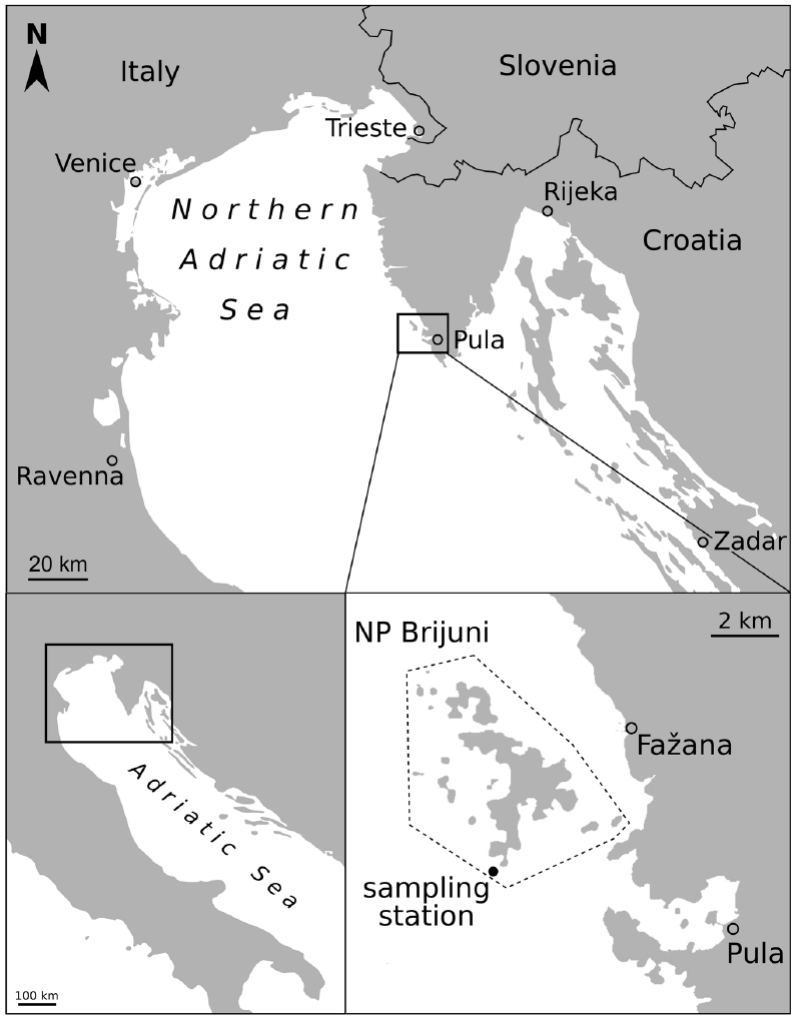
Map of the Brijuni Islands, Croatia, and exact location of the sampling station within the marine protected area of the national park Brijuni (NP Brijuni). The grey dotted line marks the boundaries of the national park.

Sampling took place in the waters of the Brijuni Islands National park, which is situated close to the SW coast of the Istrian peninsula in Croatia (Figure 1). The archipelago is spread across a total area of 7.42 km^2^ with two major islands, Mali Brijun and Veliki Brijun (Fatovic-Ferencic, 2006; Šoštarić and Küster, 2001). The first major anthropogenic impact on these islands occurred in the late 19th century when swamps were dewatered to promote tourism (Bralic, 1990; Šoštarić and Küster, 2001). In the 20th century, the area became a presidential residence and was declared a national park in 1983. Thus, its land and waters remained largely excluded from extensive commercial and public use in the late 20th century (Fatovic-Ferencic, 2006).

In summer 2013, at a water depth of 44 m (coordinates: 44.8857667 N and 13.747E), four sediment cores (length 160 cm, diameter 9 cm) were extracted with the Slovenian research vessel *Manta Bianca* using an UWITEC piston corer with hammer action (Gallmetzer et al., 2016). The core M44 selected for the molluscan community investigation was sliced directly on board into 38 subsamples (2 cm for the uppermost 20 cm and of 5 cm for the remaining 140 cm). For the purpose of this study, the data gained from the ten 2-cm slices were merged into five 4-cm slices. Grain size distribution and the concentrations of several heavy metals, nutrients and organic pollutants were measured in the second and third core (cores M45 and M46). Grain size analysis was performed by means of a sedigraph (SediGraph III 5120 Particle Size Analyzer) for the small fractions (<63 µm) and by dry sieving for fractions from 63 µm to > 1 mm. Four sediment types were defined: clay (<2 µm), silt (2–63 µm), sand (63 µm–2 mm), and the fraction > 2 mm, which mostly consisted of biogenic material. Elemental concentrations (Hg, Cu, Cr, Ni, Pb, As, Cd, Zn, Mn, Fe) were estimated with inductively coupled plasma atomic emission spectrometry (ICP-AES), organic pollutants (PCBs, PAHs) with liquid chromatography, and nutrients (N, C, TOC) with the elemental analyzer CHN 2400, according to the protocol in Vidović et al. (2016). They were determined in the core layers: 1 cm, 5 cm, 9 cm, 24 cm, 46 cm, 69 cm, 85 cm, 105 cm, 126 cm and 151 cm. In order to compare and classify the measured concentrations, we used screening levels developed by the National Oceanic and Atmospheric Administration (NOAA) of the US Department of Commerce, which represent different toxicity gradients of marine sediments. Threshold Effects Levels (TEL), Effects Range Low (ERL), Probable Effects Levels (PEL) and Effects Range Median (ERM) (ordered by increasing toxicity) are benchmarks based on databases of marine sediment chemistry and sediment toxicity bioassay data that define toxic effects (Buchmann, 2008).

### Dating of shells

Core chronology was based on the ^14^C-calibrated amino acid racemization (AAR) of 305 specimens of the bivalve *Timoclea ovata* collected in the core M44. Out of 305 specimens, 13 dead shells were selected for AMS ^14^C dating. Up to 30 specimens (complete valves or fragments with umbo preserved) of *T. ovata* were randomly selected from each of 13 more or less evenly spaced, 4- or 5-cm-thick intervals covering the whole core. *T. ovata* does not occur in the shallowest habitats and thus may not accurately date the onset of the shelf flooding at Brijuni. Therefore, four additional shells of large-sized bivalves (*Arca noae, Glycymeris* sp., *Pecten* sp., and *Ostrea* sp.) from a shell bed at 95–120 cm in the core M44 were dated with ^14^C. Dark grey, organic-rich muds with plant remains immediately underlie the molluscan gravels in the core M40, which are comparable to the lowermost part of the M44 core. A single plant remnant from this core was dated with ^14^C to geochronologically constrain the onset of marine sedimentation at the station Brijuni. The stratigraphic succession in our cores is subdivided into several units primarily on the basis of sedimentological and geochemical criteria. Age of these units is then determined on the basis of the 25th and 75th age percentiles of *T. ovata* and on the basis of ages of four shells of other species in the shell bed. Owing to slow sedimentation rates and mixing, age ranges assigned to the units are overlapping to some degree. We use the sequence-stratigraphic terminology of model III of Catuneanu et al. (2009) where the highstand phase terminates prior to the base-level fall (Hunt and Tucker, 1992).

In total, 17 bivalves and the single plant remnant were analysed for ^14^C at the AMS facility at the Poznan Radiocarbon Laboratory (Table 1). To avoid contamination, 30% of the outer shell mass was removed prior to AMS analysis in an ultrasonic bath and in 0.5M HCl, and treated in 15% H_2_O_2_ for 10 min in an ultrasonic bath. The remaining carbonate was dissolved with concentrated H_3_PO_4_ in a vacuum line. ^14^C was measured with a ‘Compact Carbon AMS’ (Goslar et al., 2004). Conventional ^14^C ages were calculated using correction for isotopic fractionation (Stuiver and Polach, 1977), on the basis of ratio ^13^C/^12^C measured in the AMS spectrometer simultaneously with the ratio ^14^C/^12^C. Conventional ages of bivalves were converted to calendar years using Calib7.1 (Stuiver and Reimer, 1993), the Marine13 calibration curve (Reimer et al., 2013), and a regional marine reservoir correction (∆R) in the NE Adriatic (Rovinj) equal to = -61 years (standard deviation = 50 years) (Siani et al., 2000). Conventional ages of the plant remnant were converted to calendar years with the IntCal13 calibration curve.

**Table 1.** Numerical ages of 13 dead-collected and three live-collected specimens of *Timoclea ovata* used in AMS-AAR calibration with D/L of the aspartic acid (Asp) and glutamic acid (Glu), and shells of four other bivalve species collected from the shell bed in the middle part of the core, and one plant remnant (organic-rich mud collected below the basis of the core M40) dated with ^14^C only. The specimen ID corresponds to the unique specimen identification number, Poznan ID is the unique identification number for the radiocarbon analyses from the Poznan Radiocarbon Laboratory. Calibrated age (yr) is relative to 2013 AD (the year of sampling).

Species	Specimen ID	Poznan ID	Calibration curve	Conventional ^14^C age	Conventional ^14^C age error (2 s.d.)	Calibrated age (median probability) BC/AD	Lower 95% conf. bound on calibrated age BC/AD	Upper 95% conf. bound on calibrated age BC/AD	Calibrated age (to 2013 AD)	Lower 95% conf. bound on calibrated age	Upper 95% conf. bound on calibrated age	Asp D/L	Glu D/L
*Timoclea ovata*	Piran-live collected (PII-G4–2)								1	2	0.1	0.034	0.018
*Timoclea ovata*	Piran-live collected (PII-G4–3)								1	2	0.1	0.036	0.021
*Timoclea ovata*	Piran-live collected (PII-G4–7)								1	2	0.1	0.037	0.022
*Timoclea ovata*	Bri M44–0–2–001	Poz-69868	Marine13	585	58	1656	1521	1815	357	492	198	0.110	0.045
*Timoclea ovata*	Bri M44–12–14–001	Poz-69869	Marine13	570	58	1675	1525	1831	338	488	182	0.130	0.035
*Timoclea ovata*	Bri M44–12–14–002	Poz-69870	Marine13	3370	58	−1350	−1493	−1200	3363	3506	3213	0.230	0.087
*Timoclea ovata*	Bri M44–20–25–001	Poz-69872	Marine13	835	58	1440	1328	1527	573	685	486	0.155	0.041
*Timoclea ovata*	Bri M44–20–25–004	Poz-69873	Marine13	815	58	1455	1332	1554	558	681	459	0.160	0.042
*Timoclea ovata*	Bri M44–20–25–005	Poz-69874	Marine13	2325	58	−67	−223	91	2080	2236	1922	0.206	0.064
*Timoclea ovata*	Bri M44–30–35–012	Poz-69875	Marine13	1900	58	436	277	578	1577	1736	1435	0.206	0.063
*Timoclea ovata*	Bri M44–50–55–029	Poz-69876	Marine13	2480	58	−261	−388	−106	2274	2401	2119	0.240	0.070
*Timoclea ovata*	Bri M44-130-135-002	Poz-69877	Marine13	5470	61	−3968	−4139	−3797	5981	6152	5810	0.272	0.097
*Timoclea ovata*	Bri M44-130-135-012	Poz-69878	Marine13	8360	71	−7079	−7295	−6841	9092	9308	8854	0.359	0.152
*Timoclea ovata*	Bri M44-130-135-015	Poz-69879	Marine13	5890	64	−4415	−4569	−4292	6428	6582	6305	0.289	0.110
*Timoclea ovata*	Bri M44-150-155-004	Poz-69880	Marine13	7220	64	−5794	−5941	−5662	7807	7954	7675	0.316	0.118
*Timoclea ovata*	Bri M44-150-155-006	Poz-69882	Marine13	8160	78	−6794	−7023	−6590	8807	9036	8603	0.334	0.137
*Arca noae*	Bri M44 115-120 A1	Poz-97839	Marine13	4990	58	−3453	−3603	−3339	5466	5616	5352	NA	NA
*Glycymeris* sp.	Bri M44 110-115 G1	Poz-97840	Marine13	7220	64	−5794	−5941	−5662	7807	7954	7675	NA	NA
*Pecten* sp.	Bri M44 110-115 P1	Poz-97841	Marine13	5520	61	−4027	−4213	−3905	6040	6226	5918	NA	NA
*Ostrea* sp.	Bri M44 105-110 O1	Poz-97842	Marine13	5710	64	−4240	−4356	−4050	6253	6369	6063	NA	NA
Plant	Brijuni M40 115-120 P	Poz-99289	IntCal13	9900	40	−9341,5	−9450	−9271	11355	11463	11284	NA	NA

Small portions of 305 dead shells of *T. ovata* were analysed for the extent of AAR at Northern Arizona University using reverse-phase high-pressure liquid chromatography (RP-HPLC, Kaufman and Manley, 1998). Specimens were leached 20% by weight with a dilute solution of HCl and the resulting solutions were hydrolysed at 110°C for 6 h to release amino acids from their peptide chains. Concentrations and D/L values of aspartic acid (Asp), glutamic acid (Glu), serine (Ser), alanine (Ala) were measured. Asp and Glu were used in age calibrations. Five specimens were flagged as outliers described by Kosnik and Kaufman (2008) and removed from analyses.

**Figure 2.**
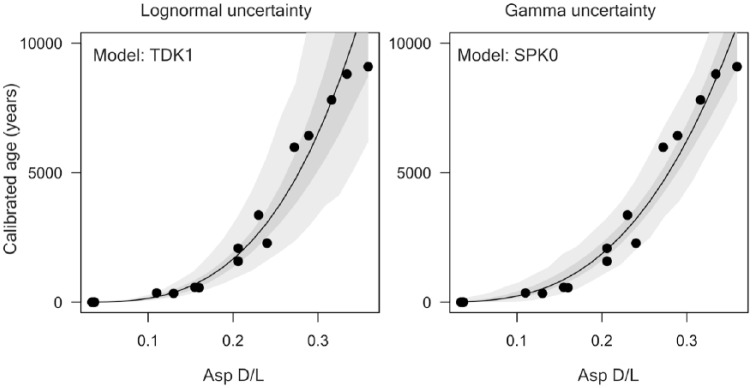
Relations between Asp D/L and ^14^C calibrated shell ages for *Timoclea ovata*, assuming log-normal and gamma distributions for the residuals, including data from live-collected specimens as calibration data points. Light-grey envelopes correspond to 95% prediction intervals for the age of a given specimen; dark-grey envelopes correspond to 95% confidence intervals for mean age.

We calibrated the rate of AAR in *T. ovata* on the basis of 13 dead shells and three live-collected shells using the Bayesian model fitting procedures according to Allen et al. (2013). Asp and Glu D/L values were fit using four mathematical functions to model the relation between age and D/L values, and two uncertainty models (lognormal and gamma) using R language (R Core Team, 2013). The four mathematical functions include time-dependent rate kinetics (TDK), constrained power-law kinetics (CPK), simple power-law kinetics (SPK), with and without fitting a non-zero initial D/L. The combination of two amino acids, two uncertainty models, and eight fitting functions gives 32 different age models. The time-dependent reaction kinetic model (TDK, Allen et al., 2013) for Asp D/L was the best model with the smallest Bayesian information criterion (BIC) (Figure 2, Table 2), with the initial D/L value estimated from data (TDK1). This model was used in the final calibration. The calibration equation is a*arctanh ((DL - DL_0_)/(1 - DL*DL_0_))^*b*, where DL is Asp D/L, DL_0_ is Asp D/L at 0 years (here, 0.02071817), and *a* = 257268.1 and *b* = 2.9614. The uncertainty is defined by the log-normal distribution, with the mean equal to age estimate and the variance equal to 0.0605. AAR-calibrated calendar ages are set relative to the year of the collection (2013 AD = year zero). Age and amino acid data are available in Supplementary Table 1, available online.

**Table 2.** Calibration statistics for the rate of amino acid racemization (AAR) based on paired AAR and radiometric analyses of *Corbula gibba* and two models of uncertainty, showing models with Bayes information criterion (BIC) values less than 6 units relative to the model with minimum BIC. Explanations: APK = apparent parabolic kinetics; CPK = constrained power-law kinetics; SPK = simple power-law kinetics; TDK = time-dependent reaction kinetics; 0 = the initial D/L value is fixed at zero; 1 = the initial D/L value is estimated from data, *a*-*d* -model parameters, R0 - the D/L value at time 0.

Amino acid	Model	ln(a)	ln(b)	c	R0	ln(d)	BIC	ΔBIC
Gamma uncertainty								
Asp	SPK0	12.333	1.094	NA	0.0000	4.620	224.19	0.00
Asp	TDK0	12.080	1.052	NA	0.0000	4.608	224.48	0.28
Glu	TDK1	12.731	0.542	1.089	0.0155	4.722	225.53	1.34
Glu	APK1	13.305	NA	0.392	0.0117	4.963	225.96	1.77
Asp	SPK1	12.341	1.095	−17.697	0.0000	4.611	227.00	2.81
Asp	TDK1	12.094	1.055	−15.839	0.0000	4.627	227.22	3.03
Glu	SPK1	13.206	0.720	1.551	0.0169	5.201	229.03	4.84
Lognormal uncertainty								
Asp	TDK1	12.458	1.086	0.278	0.0207	−2.805	211.73	0.00
Asp	SPK1	13.281	1.300	1.381	0.0312	−2.500	217.14	5.41

### Sorting, identification, and counting of shells

For the investigation of the molluscan shell record, the sediment samples were washed through a 1-mm sieve and dried. The retained material was sorted under a stereomicroscope, and molluscs were identified to species level using taxonomic literature (Cossignani and Ardovini, 2011; Cossignani et al., 1992; Gofas et al., 2011a, 2011b; Huber, 2010). The final number of bivalve individuals was computed by summing up the number of double-valved specimens with the higher number of either right or left valves. Gastropod shells were counted as individuals if at least the apex was preserved, and polyplacophorans were counted as the number of plates divided by eight.

### Functional groups

Molluscan species were categorized according to (1) feeding guild, (2) organism-substrate relation, (3) host association, and (4) weed association (Table 3). The categories and subcategories were treated separately in the analyses. Categorization follows Cachia et al. (1996), Chemello and Milazzo (2002), Morton and Dinesen (2011), Oporto et al. (2012), Rinaldi (2012), Rueda et al. (2009), Werner (1953, 1959), Beesley et al. (1998), Borja et al. (2000), Gofas et al. (2011a, 2011b), Haydar (2010a, 2010b), Huber (2010), Koulouri et al. (2006), and Oliver et al. (2016).

**Table 3.** Functional categorization used to characterize the ecology of mollusc species. Subcategories may not appear in figures but are listed here to illustrate the spread of the main categories.

feeding guild	subcategories	comments
carnivore	browsing	feeding on immobile animals such as bryozoans
	micro-carnivore	feeding on protozoans
	macro-carnivore	predators
	parasitic	living as parasites in or attached to other animals
chemosymbiotic		feeding on metabolic products of endochemosymbiotic bacteria
detritivore	on the surface	feeding on particles at the sediment surface
	sub-surface	feeding on particles below the sediment surface
grazing		feeding on detritus and micro-epigrowth (microalgae, foraminiferans)
filter-feeding		feeding on suspended particles
herbivore	micro-herbivore	feeding on microalgae
	macro-herbivore	feeding on seagrass and macroalgae
scavenging		feeding on decaying organic matter
**organism-substrate relation**	**subcategories**	**comments**
epifauna		living on the substrate (either on soft or hard bottoms)
epifauna on vegetation		living on seagrass or algae
infauna		living in the substrate (either in soft or hard bottoms)
semi-infauna		semi-burrowed species
epibionts and ectoparasites		living in a symbiotic or parasitic relationship, using their hosts as ‘substrate’
nestler		living in a byssus nest in soft, gravelly or hard substrate
terrestrial and freshwater		living on land or in freshwater
**host association**	**subcategories**	**comments**
free-living		no association
associated with host	specific associations	associated with one or several hosts belonging to the following taxa: sponges, crustaceans, echinoderms, ascidians, molluscs, polychaetes, cnidarians; or host(s) unknown
**weed association**	**subcategories**	**comments**
no weed association		living on other substrates
living on weeds	strictly seagrass	living exclusively on seagrass (eg *Posidonia oceanica, Cymodocea nodosa*)
	seagrass and algae	living on algae and/or seagrass
	mainly seagrass	frequently associated with seagrass and its rhizome layer, but also found on other substrates

### Statistical analysis

Species diversity is expressed as raw number of species and as the effective species richness, that is, the exponential of Shannon’s H (exp (H)). All values reported are means with 95% confidence intervals (1000 resampling iterations, randomly drawn without replacement). Differences in diversity between the six stratigraphic units defined on the basis of lithologic and geochemical composition were tested by a Wilcoxon rank test. PERMANOVA was used to assess differences in community structure between the six stratigraphic units on the basis of species composition and on the basis of functional composition related to (1) feeding guilds, (2) organism-substrate relation, (3) weed-, and (4) host-association. The uppermost unit (0–8 cm, last 200 years) was excluded from analyses due to insufficient sample size (minimum N = 50). Sample size of other molluscan assemblages varies between 133 and 1515 specimens. To analyse gradients in community composition, we performed a non-metric multidimensional scaling (NMDS) based on Bray-Curtis distances and square-root-transformed relative abundances. The influence of environmental variables on the composition of molluscan assemblages was explored by redundancy analysis (RDA) using a forward-model-selection approach. Clay, gravel, water depth, total nitrogen, as well as the heavy metals Hg, Pb, As, and Cd were used for the analysis. The variable with the highest adjusted R^2^ is added at each step of the model selection and its significance calculated using a permutation test (10,000 permutations) (Blanchet et al., 2008). This allows identifying the subset of environmental variables with the strongest influence. All statistical analyses were performed in R 3.2.1 (R Core Team, 2014) using the vegan package (Oksanen et al., 2015).

## Results

### Stratigraphic section and geochronology

The lowermost part of the core M40 directly overlies an organic-rich clay rich in plant debris, indicating the transition from terrestrial to marine conditions. The age of the plant remains (Table 1) is in accord with sea-level curves estimated for this region (Antonioli et al., 2007), indicating that the flooding occurred about 11,000 years ago. We distinguish six stratigraphic units that differ in sediment grain size, sediment and geochemical composition, and preservation (in terms of dominant components such as molluscs, bryozoans, and coralline algae). They include unit 1 formed by coarse-grained, loosely packed and moderately sorted molluscan sands at 120–160 cm, unit 2 formed by densely packed and poorly sorted molluscan gravel (shell bed) at 90–120 cm, sharply delimited by an abrupt increase in abundance of large molluscs on the bottom and by an abrupt decline in their abundance on the top, unit 3 formed by poorly sorted and loosely packed bryozoan-molluscan muddy sand at 50–90 cm, unit 4 formed by poorly sorted and loosely packed bryozoan-molluscan sandy mud at 20–50 cm, unit 5 formed by sandy mud with dispersed bioclasts at 8–20 cm with high concentrations of pollutants (PCB and PAH), and unit 6 formed by sandy mud with coralline algae and dispersed larger shells with high concentrations of total nitrogen at 0–8 cm.

**Figure 3.**
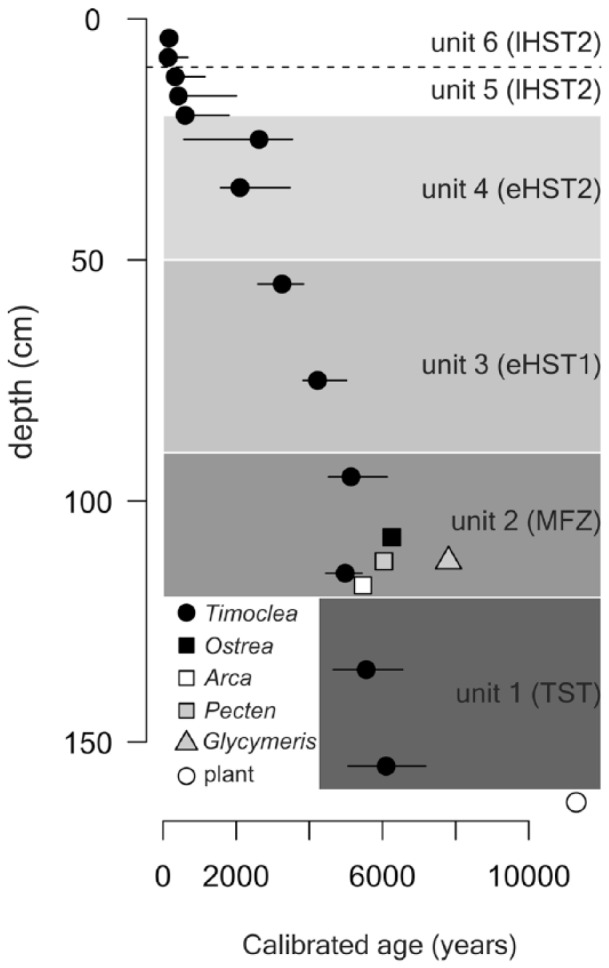
Stratigraphic distribution of shell ages of *Timoclea ovata* in the Brijuni core M44 (median age in black circles, and the 25th and 75th age percentiles corresponding to minima and maxima), and radiocarbon ages in *Ostrea* sp., *Arca noae, Pecten* sp., *Glycymeris* sp., and a single plant remain. The difference between the 75th and 25th age correspond to inter-quartile age ranges.

In terms of the 25th and 75th age percentiles, shell ages are younger than 370 years in unit 6, range between 200–1700 years in unit 5, 1500–3600 years in unit 4, 2900–4600 years in unit 3, 4500–7500 years in unit 2, and 4800–7500 years in unit 1. Median ages of *T. ovata* decline almost monotonically from 140–160 years in the upper part (sediment depth 0–8 cm) to 6100 years in the deepest part at 150–155 cm, with the upper age quartile equal to 7180 years and maximum age equal to 10,600 years (Figure 3). The median age of *T. ovata* in two increments in the shell bed (unit 2) is 5000–5100 years. However, bivalve shells of four large-sized species that represent the bulk of the shell bed at 95–120 cm are older (5500–7800 years) and together indicate that the shell bed was formed ~5000–8000 years ago.

Raw inter-quartile age ranges of *T. ovata* in 4 cm increments reach 280–650 years in unit 6 and 1000–3000 years in units 1–5. The correction for calibration error using lognormal distribution (according to Dominguez et al., 2016) disproportionately reduces the inter-quartile age ranges in the lowermost units: inter-quartile age ranges corrected for calibration error reach 230–600 years in unit 6, 1000–2000 years in unit 4–5, and 400–1300 years in unit 3–5, and smaller, centennial ranges (200–250 years) in units 1–2. However, in spite of the correction, *T. ovata* death assemblages in 4-cm increments are time averaged from several centuries up to ~2000 years. Although geochronological data indicate that these units differ in median age, these scales of time averaging generate substantial stratigraphic overlaps (Figure 3). In spite of these overlaps, the six units can be assigned to sea-level phases independently reconstructed in this region (Antonioli et al., 2007). Unit 1 largely corresponds to the transgressive phase prior to the maximum ingression (120–155 cm), unit 2 approximately coincides with the transition between the late transgressive and early highstand phase (maximum-flooding zone) that occurred ~7500–5500 years ago (90–120 cm), units 3–4 with the early highstand phase (20–85 cm), and units 5–6 with the late highstand phase when the sea-level was stabilized ~2000 years ago (0–20 cm).

### General trends in sediment composition

**Figure 4.**
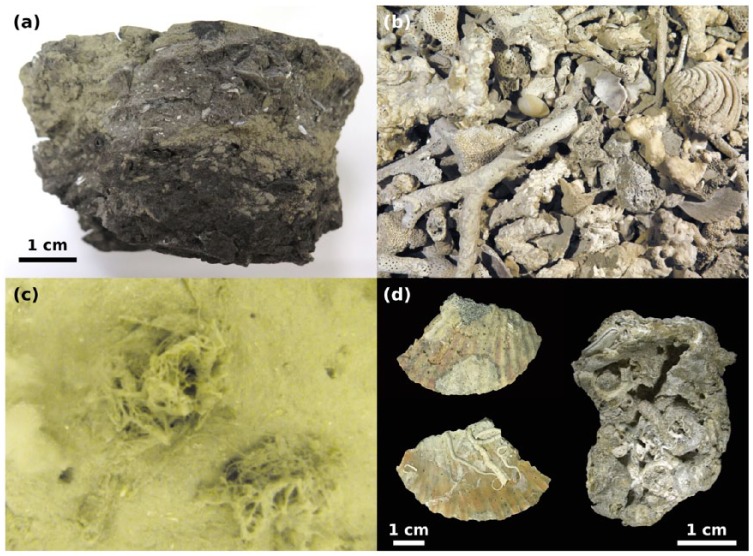
(a) Paralic layer from the lowermost core increment originating from the period before the Holocene transgression, (b) bryozoan-molluscan gravel dominating the upper core parts until 95 cm depth, (c) muddy sediment surface at the sampling station with two bryozoan colonies covered by mucilage, and (d) pectinid and oyster shells from the macroid layer overgrown by serpulid tubeworms and laminar bryozoans and heavily bioeroded by boring sponges; photographs by Ivo Gallmetzer and Alexandra Haselmair.

**Figure 5.**
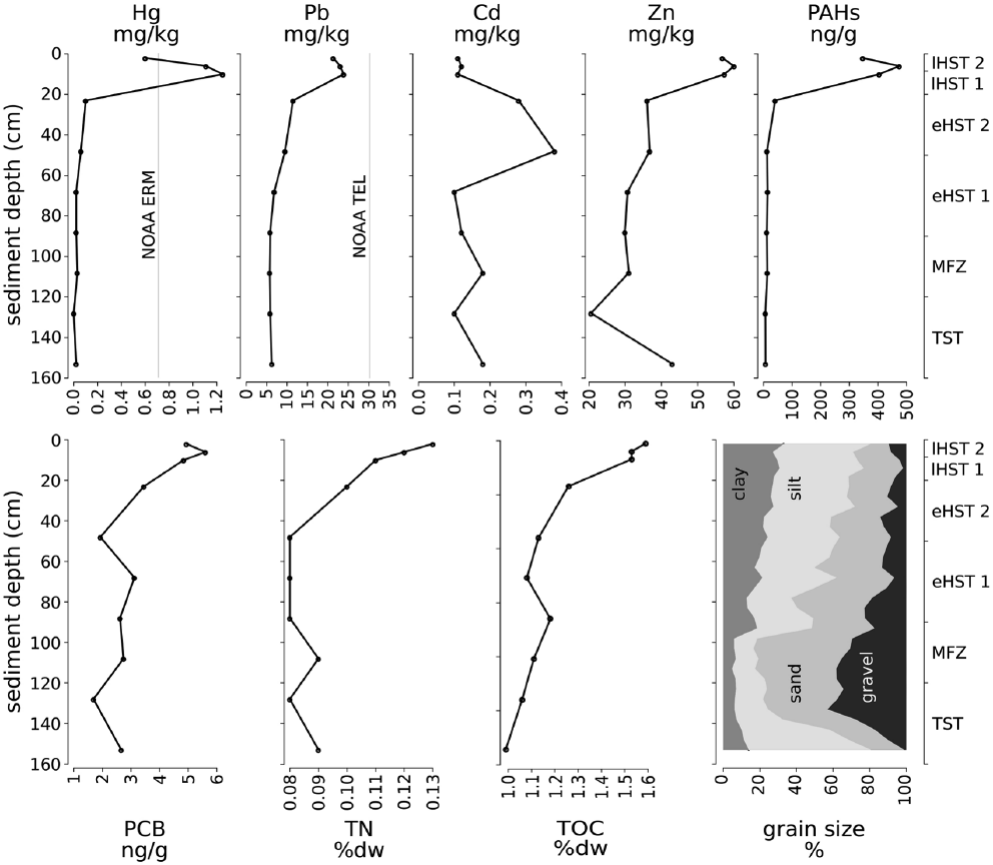
Concentrations of heavy metals and organic pollutants along the core. Hg, Pb, Cd, and Zn in mg/kg; PAH and PCB in ng/g; and TOC and TN in percent dry weight. Dotted lines boundaries of age units. Hg concentrations exceed NOAA ERM benchmark (grey line).

At the bottom of the core, dark grey, organic-rich clays with frequent plant remains mark the transgression from a paralic to a marine environment (Figure 4). Unit 1 is formed by molluscan muddy sand with a mixture of stained and pristine bioclasts. Higher up, the skeletal gravel and sand fraction increase steeply and reach a maximum of ~80% in unit 2 at 100 cm. This peak corresponds to a ~25-cm-thick, densely packed and poorly sorted bivalve shell bed with high abundance of encrusting organisms. The units 3–6 contain abundant bryozoans and molluscs and show a fining-upward trend, leading to a high proportion of clay and silt (~80%) in surface sediments (Figure 5). The heavy metals Hg, Pb and Zn attain maxima in the upper 20 cm, while Cd peaks at 50 cm at the boundary between units 3 and 4. Only Hg exceeds NOAA ERM levels during unit 2. The trend of Cr, Cu and Ni is closely correlated with the fine sediment fraction (silt and clay), while no correlation is evident for the other metals. Fe and Al show opposite trends: Fe concentration reaches 19.5 g/kg in the deepest subsample (150–152 cm), while Al concentration peaks at a depth of 23–25 cm (17.6 g/kg). High concentrations of polycyclic aromatic hydrocarbons (PAHs) occur in the uppermost 12 cm, with a maximum of 475 ng/gr at 5 cm. Polychlorinated biphenyls (PCBs) also reach their maximum (6 ng/gr) at 5 cm core depth. In the rest of the core, their concentrations are very low, in the case of PAHs close to zero. TOC and total nitrogen increase up-core (Figure 5).

### Molluscan assemblages

**Figure 6.**
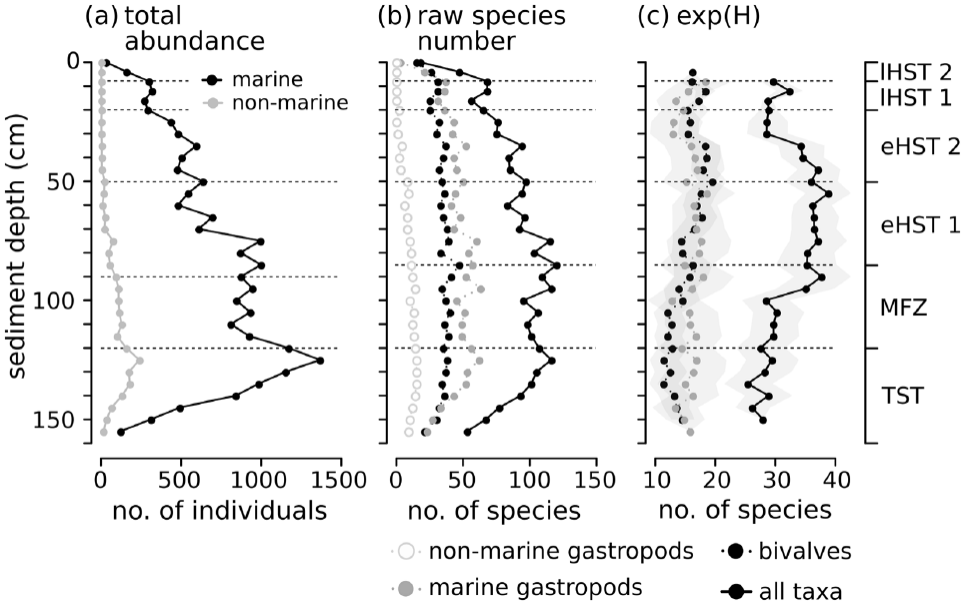
Abundance and diversity of molluscan taxa in the sediment core along a depth (left) and time axis (right). The unit boundaries marked by dotted lines. (a) total abundance, (b) raw number of species, and (c) effective number of species (exp (H)).

**Figure 7.**
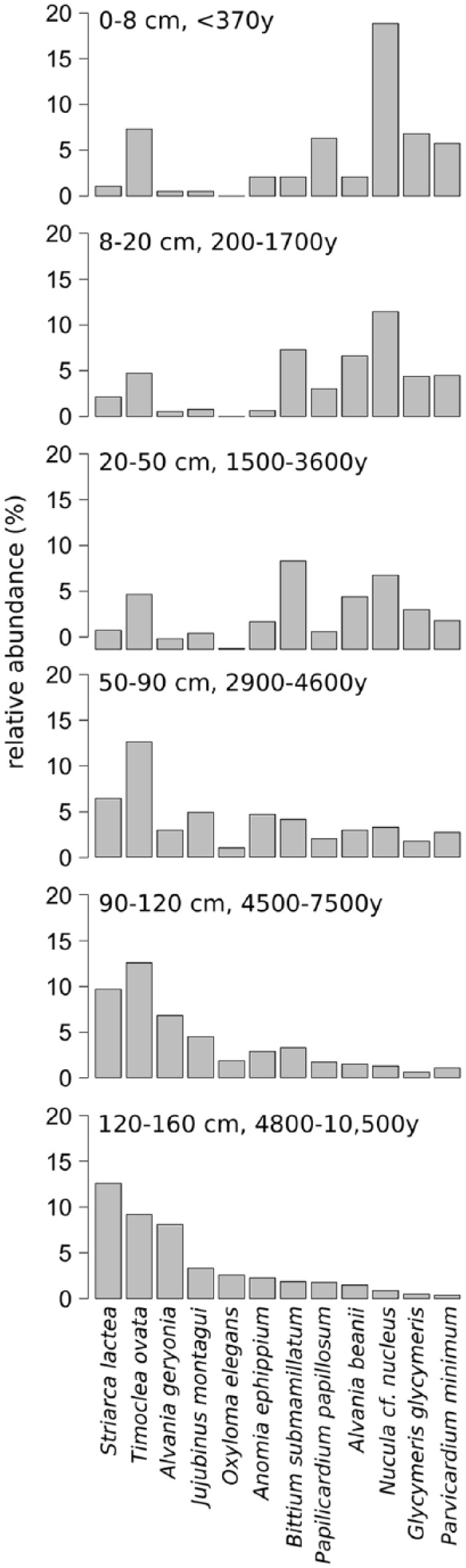
Relative abundances of the 12 most abundant species in each unit. *Oxyloma elegans* is a freshwater-associated land snail, all other species are marine. Age ranges correspond to the 25th and 75th age percentiles and refer to calibrated years before 2013 AD.

Throughout the core, 23,012 individuals representing 229 mollusc species from 106 families were identified, including 24 non-marine gastropod species, which are very abundant in the deeper parts until 70 cm depth. Gastropods account for 149, bivalves for 69, polyplacophorans for 8, and scaphopods for 3 species. Gastropods are more abundant than bivalves in most of the core, but are absent in the uppermost 8 cm (last 200 years, Figure 6). Total abundance of individuals peaks in unit 1 at 130 cm (>5500 years ago) and strongly decreases up-core. Diversity changes are best illustrated by the effective number of species, which peaks between 35 and 90 cm core depth (2000–4500 years ago) (Figure 6). Apart from molluscan shells, the sediment core was also rich in calcareous skeletal remains, mostly of bryozoans, echinoderms, and crustaceans (Pifeas, 2017). Layer 95–120 cm was characterized by a high abundance of heavily bioeroded shells with thick calcareous encrustations, mainly by bryozoans. The six units differ significantly in species composition and abundance (Figure 7 and Table 4; unit 6 was excluded due to small sample size).

**Table 4.** Differences in species composition and functional groups between age units (tested with PERMANOVA, except for exp(H), where a Wilcoxon-rank test was applied). Significant results in bold. The 0–8 cm unit was not tested because of an insufficient number of individuals.

temporal bins		8-20 vs 20-50 cm	20-50 vs 50-90 cm	50-90 vs 90-120 cm	90-120 vs 120-160 cm	8-50 vs 50-90 cm
species	F	2.08	4.63	3.99	2.07	4.61
	Pr(>F)	**0.027**	**0.001**	**0.004**	**0.007**	**0.001**
feeding guild	F	1.37	7.84	9.99	4.84	6.50
	Pr(>F)	0.21	**0.005**	**0.001**	**0.004**	**0.004**
organism-substrate relation	F	0.98	8.77	6.31	4.77	9.45
	Pr(>F)	0.45	**0.001**	**0.002**	**0.008**	**0.001**
host association	F	3.2	2.23	1.44	2.37	1.30
	Pr(>F)	**0.011**	0.077	0.26	**0.037**	0.312
weed associoation	F	2.52	9.71	13.04	1.50	7.0
	Pr(>F)	0.102	**0.003**	**0.001**	0.206	**0.004**
exp(H)	p-value	0.905	**0.02**	**0.029**	**0.005**	**0.003**

**Figure 8.**
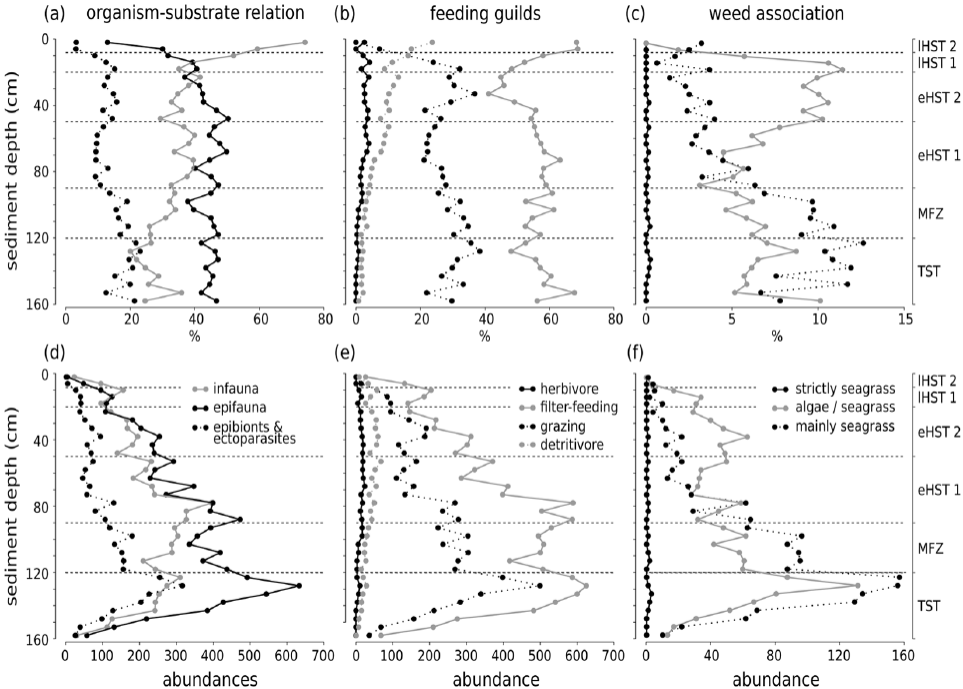
Up-core shifts in the dominant substrate relations, feeding types, and weed associations since the Holocene transgression, plotted for relative (a-c) and absolute abundances (d-f). Dotted lines mark unit boundaries.

**Figure 9.**
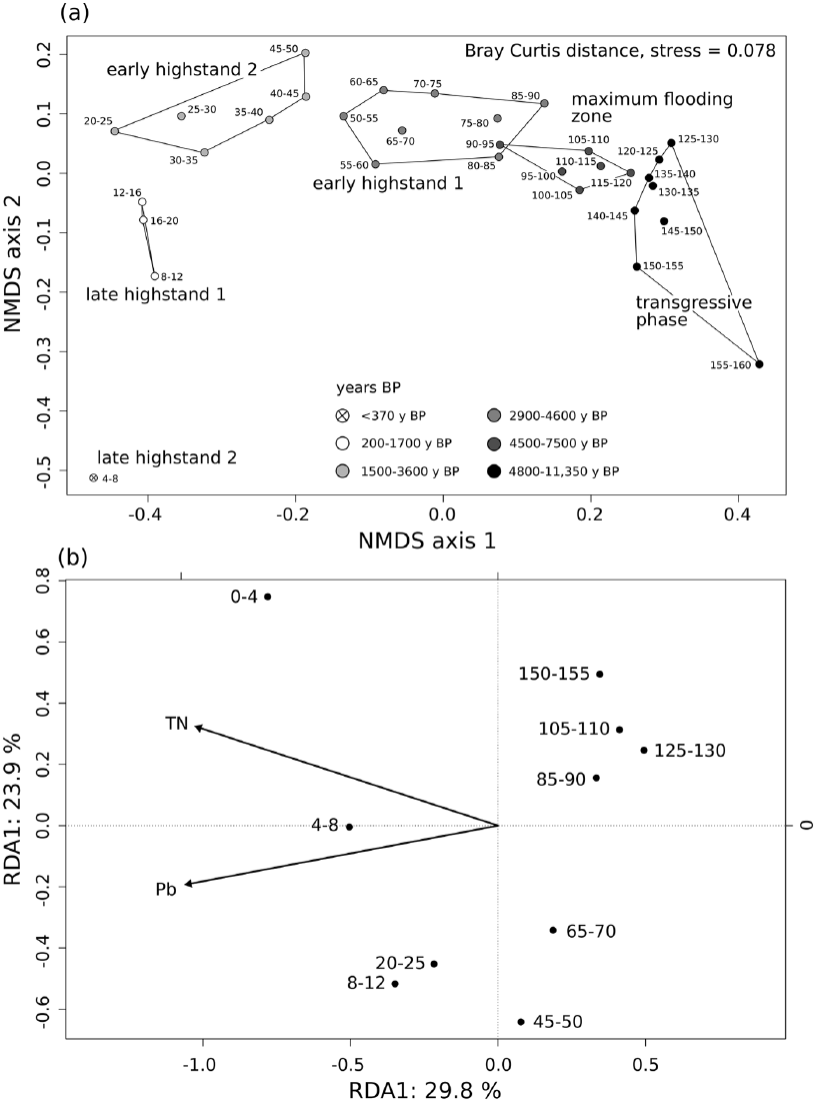
(a) NMDS plot of all determined marine mollusc species and (b) redundancy analysis, with Pb and total nitrogen (TN) identified as the main environmental drivers of molluscan community change.

Throughout the core, epi- and infaunal life habits dominate, and filter-feeders are the most important feeding guild. Epibionts and ectoparasites are more abundant in deeper sediment layers. The relative (and absolute) abundance of detritivores increases up-core while the abundance of seagrass-associated species decreases (Figure 8). The bivalves *T. ovata* (infaunal filter feeder), *Anomia ephippium* and *Striarca lactea* (epifaunal filter-feeders), and the herbivorous gastropods *Alvania geryonia* and *Jujubinus montagui* dominate in the three lowermost units of the core from 155 cm to 50 cm core depth. With the exception of *T. ovata*, these species experience a strong up-core decrease in abundance. The grazing gastropod *Bittium submamillatum*, on the other hand, peaks in abundance between 20 and 50 cm. In general, molluscs specialized on coarse-grained or hard substrate (*S. lactea, A. ephippium*) decrease up-core with an increase of the mud proportion. In contrast, abundances of infaunal filter-feeders (e.g. *Corbula gibba, Glycymeris glycymeris, Parvicardium* spp.) and detritivores such as Nuculidae and Nuculanidae (*Lembulus pella, Saccella commutata, Nucula nucleus*) increase (Figure 8). Differences in functional composition between stratigraphic units are significant for most consecutive units, except for units 2 and 3 (8–20 cm and 20–50 cm) (Table 4). The NMDS analysis of all marine species shows that the assemblages are stratigraphically aligned along axis 1 (Figure 9a). This pattern is similar in NMDS analyses based on the proportional abundances of feeding guilds, substrate groups, and weed-, and host associations (see online data set). In all ordinations, assemblages are arranged into six relatively distinct groups along the first axis, corresponding to the six units (Figure 9a).

### Unit 1 (120–160 cm, 4800–10,500 years)

A relatively rapid shift in grain size occurred in this unit, from 80% clay and silt at 165 cm to 80% sand (mostly consisting of molluscs), at 120 cm (Figure 5). Total abundance of mollusc shells is low in the deepest layers, but increases steadily towards the upper unit boundary. This unit contains the sample with the highest abundance (1557 specimens at 125–130 cm) of molluscs in the entire core. The raw number of species shows an up-core increase, whereas the effective number of species remains more or less stable (Figure 6). The most dominant species are *S. lactea* and *T. ovata*. The two grazing gastropods *A. geryonia* and *J. montagui* as well as the pulmonate land snail *Oxyloma elegans* are also abundant (Figure 7). Land- and freshwater species are numerous together with a wide range of marine epifaunal filter-feeders and grazers. A strong increase in the abundance of epifaunal filter-feeders and grazers is observed in this unit, from zero to >500 individuals. A similar trend is documented for algae- and seagrass-related species (Figure 8).

### Shell bed (90–120 cm, 4500–7500 years)

This unit corresponds to a marked shell bed with large-sized, randomly oriented bivalves. Grain size composition shifts from 80% sand and gravel to 50% silt and clay. Total abundance and species richness peak in this unit (Figure 6). *T. ovata* is the most abundant species, followed by *S. lactea, A. geryonia, J. montagui* and the epifaunal grazer *B. submamillatum*. The effective number of species increases suddenly at 100 cm sediment depth and the entire unit has significantly lower diversity than unit 1 (Wilcoxon rank test: p = 0.002). The assemblage consists mainly of epifaunal species, and grazing is the most important feeding strategy in gastropods.

### Unit 3 (50–90 cm, 2900–4600 years)

The sediment composition in this unit formed primarily by bryozoan muddy sand shifts towards higher proportions of silt and clay (60%), accompanied by a decrease in total molluscan abundance. Diversity (exp(H)) remains high (Figure 6) but is still significantly lower than in unit 2 (Wilcoxon rank test: p = 0.02). Although *T. ovata* is the bivalve species with the highest abundance (relative and absolut), epifauna (*S. lactea, A. ephippium, J. montagui, B. submamillatum*) is dominant (Figure 7). Filter-feeders still represent the most important feeding guild, but show a slight proportional decrease towards the upper boundary while detritivores slowly increase. Molluscs with a strong association to seagrasses are replaced by taxa with no specific weed preference (Figure 8).

### Unit 4 (20–50 cm, 1500–3600 years)

The fining-upwards trend in sediment grain size (from 21% to 27% of clay) persists within this unit formed by bryozoan-molluscan sandy mud and characterized by a marked community shift in both species composition and functional groups. Total abundance and diversity (exp(H)) strongly decrease (Wilcoxon rank test: p = 0.02). This change occurs abruptly at about 30 cm (~2000 years ago, Figure 6). Concomitantly, epifauna proportionally decreases in favour of infauna, and relative abundances of detritus feeders and grazers rise (Figure 8). The most abundant species is *B. submamillatum*, closely followed by the soft bottom infaunal filter- and detritus feeder *N. cf. nucleus.* Abundances of other species also change markedly, with *T. ovata* decreasing and *Alvania beanii* and the filter-feeder *Glycymeris glycymeris* increasing upwards (Figure 7).

### Unit 5 (8–20 cm, 200–1700 years)

Clay and silt constitute 78% of the sediment in this unit formed by rhodolithic-molluscan-bryozoan-sandy mud with higher concentrations of pollutants. Total abundances of molluscs are low in comparison to former units. Species richness and effective number of species reverse and increase again (Figure 6). Infaunal bivalves and epifaunal gastropods characterize this unit. *N. cf. nucleus* is the most abundant species in the uppermost 20 cm, including unit 6 (Figure 7). Filter-feeders and detritivores increase in abundance while grazers strongly decrease. The epifaunal grazers *B. submamillatum* and *A. beanii* are still important, but infaunal filter-feeders such as *T. ovata, Parvicardium minimum* and *G. glycymeris* become more abundant (Figures 7 and 8).

### Unit 6 (0–8 cm, < 370 years)

The sediment in this unit, whose assemblage is strongly segregated from the rest of the core in ordination space, is formed by rhodolithic–molluscan–bryozoan sandy mud with organic enrichment and consists mainly of pelitic material (Figure 5). Abundances and diversity (exp(H)) drop to minimum values (Figure 6). Soft bottom filter-feeding and detritivore bivalves are dominant. *N. cf. nucleus* is by far the most common species and other infaunal or semi-infaunal species such as *T. ovata, Papillicardium papillosum, P. minimum*, and *G. glycymeris* are also relatively frequent (Figures 7 and 8).

### Relation between environmental parameters and community change

The RDA shows that Pb (F = 5.424, p = 0.002) and TN (F = 2.1638, p = 0.014) have highly significant effects on the variation in the species composition of molluscan assemblages. However, clay content, Hg, Zn, PAHs and PCBs significantly correlate with concentrations of Pb and TN. Therefore, effects of these variables can be masked by the effects of Pb and TN (Figure 9b).

## Discussion

Our analyses show that the sediment cores collected at 44 m water depth from the Brijuni Islands reach back to the Holocene transgression about 11,000 years ago. They capture the transgressive phase coupled with shoreface erosion and reworking, followed by the establishment of epifaunal bivalve-dominated biostromes during the late transgressive/early highstand transition (maximum-flooding zone), by the shift to low-energy bryozoan-dominated biostromes during the highstand phase, and finally by strong environmental changes triggered by human impact mainly during the last century. Although molluscan assemblages are time-averaged at multi-centennial to millennial scales (Figure 3), the changing species composition gives insight into the environmental shifts occurring over longer, Holocene time-scales. The emergence of epifaunal molluscs associated with seagrasses or algae point to a habitat with marine vegetation during the transgressional phase. During highstand, bryozoans began to dominate and shape the benthic community at the expense of epifaunal molluscs. Finally, the up-core increase of fine sediments led to a dominance of infaunal molluscs.

### Transgressive phase (unit 1): Mixing of freshwater and marine assemblages during the Holocene transgression

We infer that the molluscan assemblages during this phase largely formed since the flooding ~11,000 up to 7000–8000 years ago. The Holocene boundary with the underlying plant-rich muddy layer (Figure 4a) and the high abundance of land and freshwater gastropod species in the deepest increments are indicative of the terrestrial, freshwater-dominated environments (marshlands, swamps, and estuaries) occurring on the northern Adriatic coastal plains prior to the formation of barrier-lagoon systems during the marine transgression (Bortolami et al., 1977; Correggiari et al., 1996b; Trincardi et al., 1994). The mixture of land and freshwater snails and marine molluscs in unit 1 documents the transition from a terrestrial to a shallow, nearshore to upper shoreface environments on a sandy, high-energy seafloor (Figure 7). Lithologic changes and paleoecological and taphonomic attributes of assemblages in unit 1 in the lowermost core part are closely related to the flooding, initial deposition in nearshore barrier-lagoon system (comparable to similar systems located at 42 m water depth and flooded ~10,500 years ago, Storms et al., 2008) and subsequent reworking and cannibalization of nearshore deposits due to high-energy shoreface erosion. These processes led to a mixture of freshwater, brackish, and marine species in molluscan gravels preserved in unit 1 (i.e. transgressive lags or transgressive sand sheet, Cattaneo and Steel, 2003). The transgressive lag deposits consisting of molluscan shell hash, frequently with worn and stained preservation, indicates long residence times and repeated reworking (as indicated by mixing of freshwater and marine species and by interquartile age range of *T. ovata* ages, Figure 3). Therefore, although the barrier-lagoon system was overstepped and preserved during the shoreface retreat in some portions of the northern Adriatic Sea (Storms et al., 2008), this system was probably reworked and cannibalized into molluscan lag deposits at Brijuni. The high abundance of grazing gastropods and a dominance of epifaunal species such as *Striarca lactea* and species associated with seagrass and algae such as *Bittium latreilli, Bittium reticulatum, Alvania spp., Rissoa spp., Tricolia pullus* and *Jujubinus striatus*, point to abundant seafloor vegetation and clear water conditions, with the development of seagrass meadows and macroalgal stocks.

### Maximum-flooding zone (unit 2): Establishment of diverse epifaunal biostromes

The sediment is predominantly formed of gravel and sand-sized shells and shell fragments of large-sized scallops, glycymeridids, arcids, and oysters. The effective number of species peaks in this unit (Figure 6) indicating the establishment of a highly diverse marine fauna. In the late phase of sea level rise, the habitat changed from macrophyte-dominated towards biogenic hard bottoms either in the form of extensive bivalve biostromes (similar to some degree to an extinct, oyster-dominated shell bed documented by Mautner et al. (2018) in the southern Gulf of Trieste) or in the form of isolated multi-species clumps as present today in some parts of the northern Adriatic (Fedra et al., 1976). High abundances of epifaunal carnivorous molluscs (Mangeliidae, Conidae, Raphitomidae) as well as parasites on sponges (Triphoridae) and sea anemones (Epitoniidae) further demonstrate the presence of habitat-forming aggregations of large suspension-feeding invertebrates. Most molluscan shells (oysters, pectinids, arcids, and glycymeridids) are heavily bored and encrusted by bryozoans (Figure 4d), indicating low sedimentation rates and substrate stability, providing ideal conditions for settlement of epibionts and encrusters. The temporal coincidence of this shell bed with the timing of fastest sea level rise ~7000–6000 years ago and high degree of encrustation and bioerosion indicates the connection between shoreline retrogradation, reduced sediment supply, and shell-bed formation, with potential for taphonomic feedback. Similar examples were described from maximum-flooding zones of cores collected in offshore locations off the Po Delta (Scarponi et al., 2017b). However, grazing species (*B. submamillatum*, a gastropod feeding preferentially on micro-epigrowth of various substrates) and macro-herbivores (*A. geryonia, Smaragdia* sp.) are still numerous and hint at the mixture of epifaunal biostromes and patches of marine vegetation. Thus, the maximum-flooding phase was still characterized high water clarity, with high light penetration as suggested by the presence of species depending on marine vegetation (Gacia et al., 1999; Ruiz and Romero, 2003).

### Early highstand (unit 3): Algal-bryozoan meadows

The community in this section is characterized by an increasing importance of bryozoans, which make up a large part of the death assemblage (Figure 4b) (Pifeas, 2017), demonstrating a shift from vegetation-dominated communities and bivalve shell beds to bryozoan meadows. Together with the increasing siltation and sediment trapping, the shift in species composition towards higher abundance of infaunal suspension- and deposit-feeders and towards a decline of seagrass-related molluscs suggests that seagrass meadows vanished with increasing water depth, reduced light penetration and higher turbidity (Figure 8) (Abal and Dennison, 1996; Duarte, 1991). However, species associated with algae (*Pusillina* spp., *Bittium* spp., *Vexillum ebenus, Mangelia stosiciana, Haminoea hydatis*) increase in relative abundance (Figure 8). Although this change from a vegetation- to a bryozoan-dominated seafloor coincides with the beginning of human settlements, limited agriculture, and early fishing activities in this region in the mid-Neolithic (Bralic, 1990; Horvat, 2015; Šoštarić and Küster, 2001), benthic assemblages and sediment composition are still largely unaffected by human impacts. Community changes can rather be ascribed to natural long-term environmental shifts resulting from deepening (Asioli et al., 1996; McKinney, 2007).

### Early highstand (unit 4): Siltation and the growing importance of deposit feeders

The drop in mollusc abundance and the relative increase in abundance of infaunal deposit-feeders and infaunal suspension-feeders such as *G. glycymeris* (Gofas et al., 2011b) and of species without seagrass association (Figure 8) reflects the final demise of seagrass meadows. The substitution of *Alvania geryonia*, a rissoid gastropod associated with seagrass and one of the most common species in units 1–3, by its sister species *A. beanii*, a detritus-feeder and grazer (Gofas et al., 2011b), is in accord with the demise of vegetational cover on soft bottoms. Several detritivore species increase in abundance and mark a shift in functional community composition, illustrating the growing importance of detritus as food source in a more nutrient-enriched, siltier environment, as also suggested by increasing concentrations of TN (Figure 5). *Nucula cf. nucleus*, a sub-surface filter- and detritus feeder (Gofas et al., 2011a; Wood and Turner, 2012) becomes the second-most abundant species in this unit, while the infaunal filter feeder *T. ovata* decreases markedly. The most abundant gastropod, *B. submamillatum*, is a grazer on both soft and hard substrates and was therefore probably less affected by the switch from coarser to finer sediment. During this period, human settlements were growing rapidly. Fishery efforts and inland deforestation carried out by the Celts and Romans represented new impacts on the coastal environment and may have contributed to the observed species and functional turnover by enhancing sediment loads and nutrient input (Airoldi and Beck, 2007; Boero, 2001).

### Early and late anthropogenic impact (units 5 and 6): Pollution and eutrophication

The concentrations of pollutants and nitrogen content in these two units trace a growing anthropogenic impact on the sea, deriving from expanding coastal settlements, land use, industrialization, and concomitant pollution and eutrophication (Lotze et al., 2006). This impact is documented by the abrupt and steep increase in the concentrations of heavy metals (Hg, Pb, and Zn) and organic pollutants (PAHs and PCBs) (Figure 5). The most probable sources of this pollution are the adjacent port of Pula with its dockyard, and the use of the area as a naval port during the late 19th and most of the 20th century (Fabijanović Matijašec, 1971, 2008). The presence of these elements and organic compounds, which are unequivocally of industrial origin (Covelli et al., 2006; Rios et al., 2007; Vane et al., 2007), in sediment layers that are several centuries old is probably a consequence of sediment mixing. However, all mentioned pollutants recede again in the uppermost 5–10 cm of sediment, illustrating a trend-reversal after the late-20th century efforts to reduce environmental pollution (Lotze et al., 2011). In contrast to heavy metals and organic pollutants, the concentration of total nitrogen increases steeply and steadily up to the surface, indicating that wastewater discharge and agriculture represent the main sources for an ongoing eutrophication process in this coastal area (Howarth, 2008).

Our analyses indicate that compositional variability of molluscan assemblages is primarily related to changes in nitrogen and Pb concentration, which both correlate closely with clay content (Figures 5 and 9b). While increasing sediment siltation on the transition from the transgressive to the highstand phase can be mainly seen as a consequence of deepening and changes in circulation patterns and current regimes (Artegiani et al., 1997; Boldrin et al., 2009), during the deposition of the uppermost two core units (0–1000 years) this process was further augmented by human activities such as coastal deforestation (Kranjc, 2009) and intensified bottom trawling. Although the Brijuni archipelago is protected since 1983 (Fatovic-Ferencic, 2006), this fishing method has been widely used in the northern Adriatic since the 20th century (Bolje et al., 2014) and can be responsible for high rates of sediment resuspension (Kaiser et al., 2002; Korpinen et al., 2013; Zuschin and Stachowitsch, 2009).

The selective loss of epifaunal species is reminiscent of the loss of epifaunal communities in the southern Gulf of Trieste in the 20th century as documented for a sampling station at 20 m water depth (Mautner et al., 2018). However, on one hand, the shell bed at Brijuni was naturally replaced by bryozoan meadows owing to deepening, reduced light penetration, and increased sediment trapping. On the other hand, at the deeper Brijuni station (44 m), bryozoans and other epifaunal molluscs were also severely affected by eutrophication and pollution. At times of sampling, the sea-floor was largely formed by dead skeletal debris and isolated epifaunal clumps. Bryozoan meadows, which are still preserved at some other places off Istria (McKinney and Jaklin, 2001), were degraded and not represented by living colonies (Figure 4c). Under increasing anthropogenic pressure, the molluscan community shifted towards high abundances of infaunal deposit feeders such as Nuculidae (*N. nucleus*). Epifaunal filter-feeding species disappeared together with many carnivores, hinting at decreased water quality and changes in sediment composition (Nerlović et al., 2011). Gastropods are extremely rare in surficial sediment layers, and infaunal bivalve species sensitive to organic enrichment and pollution such as *Clausinella fasciata* (Borja et al., 2000) are also absent.

In contrast to the decline of suspension feeders, chemosymbiotic bivalves (mainly the lucinid *Myrtea spinifera*, a species not associated with seagrass meadows but adapted to life in low-sulphide sediments (Dando et al., 1985)), although present only in small numbers, were completely absent in the oldest core sections, appeared for the first time in unit 3 and then increased in unit 6. This trend can be linked to the organic enrichment of the sediment and to increasing frequency of hypoxic and anoxic events in the northern Adriatic during the last century (Djakovac et al., 2015; Giani et al., 2012; Justic, 1987). Organisms living in symbiosis with chemoautotrophic bacteria (that require anoxic conditions within the sediment) are better adapted to conditions with low concentrations of dissolved oxygen (Dubilier et al., 2008; Stewart et al., 2005) and might benefit from the disappearance of other benthic species from affected areas.

## Conclusion

The core sampled at Brijuni exemplifies the environmental transition from a terrestrial to a marine soft- and mixed-bottom habitat in the south-eastern part of the northern Adriatic Sea. Natural processes caused by sea level rise during the Holocene transgression explain changes in the composition and diversity of molluscan assemblages during the deposition of the first four units. Seagrasses and macroalgae probably characterized benthic habitats during the early transgressional phase, together with the formation of biostromes with epifaunal bivalves. Seagrass habitats disappeared with increasing water depth due to sediment siltation and reduced light penetration. Bryozoan meadows developed during the highstand phase, and molluscan community was dominated by infaunal suspension-feeders and deposit feeders, mostly bivalves. Grazing gastropods, which were deprived of their main food source, decreased strongly in abundance up-core. First human settlements appeared and small-scale deforestation and agriculture during the latest highstand phase further contributed to seafloor siltation. In contrast to the earlier core units, which were defined by large-scale oceanographic events such as sea level rise and changing current patterns, the effects of pollution and eutrophication dominate in the units 5 and 6. Therefore, humans have re-shaped the environment during the past centuries with increasing speed. The late highstand benthic assemblages with abundant bryozoans, high molluscan diversity, and abundance of soft-bottom epi- and infaunal filter feeders and algae-associated species emerge as the last baseline community largely unaffected by anthropogenic impacts and could thus be a reference point for any management or conservation efforts aiming to restore pre-impact conditions.

## Supplemental Material

Supplement_data-table_S01 – Supplemental material for Molluscan benthic communities at Brijuni Islands (northern Adriatic Sea) shaped by Holocene sea-level rise and recent human eutrophication and pollution Click here for additional data file. Supplemental material, Supplement_data-table_S01 for Molluscan benthic communities at Brijuni Islands (northern Adriatic Sea) shaped by Holocene sea-level rise and recent human eutrophication and pollution by Schnedl Sara-Maria, Alexandra Haselmair, Ivo Gallmetzer, Anna-Katharina Mautner, Adam Tomašových and Martin Zuschin in The Holocene

## Supplemental Material

Suppl_data-table_S02 – Supplemental material for Molluscan benthic communities at Brijuni Islands (northern Adriatic Sea) shaped by Holocene sea-level rise and recent human eutrophication and pollution Click here for additional data file. Supplemental material, Suppl_data-table_S02 for Molluscan benthic communities at Brijuni Islands (northern Adriatic Sea) shaped by Holocene sea-level rise and recent human eutrophication and pollution by Schnedl Sara-Maria, Alexandra Haselmair, Ivo Gallmetzer, Anna-Katharina Mautner, Adam Tomašových and Martin Zuschin in The Holocene
